# Reporting Frequency of Antipsychotics‐Induced Tardive Dyskinesia and Other Extrapyramidal Symptoms: Analysis Based on a Spontaneous Reporting System Database in Japan

**DOI:** 10.1002/npr2.70049

**Published:** 2025-09-12

**Authors:** Yosuke Saga, Hiroshi Horio, Chih‐Lin Chiang, Akihide Wakamatsu

**Affiliations:** ^1^ Medical Affairs Department Johnson & Johnson Tokyo Japan

**Keywords:** antipsychotics, extrapyramidal symptom, Japanese Adverse Drug Event Report (JADER) database, reporting odds ratio, tardive dyskinesia

## Abstract

First‐ and second‐generation antipsychotics (FGAs and SGAs, respectively) with dopamine‐antagonizing properties may cause involuntary movement‐related adverse drug reactions (ADRs). However, the risk in the Japanese population is not well characterized. In this study, we analyzed spontaneous ADR reports from the Japanese Adverse Drug Event Report (JADER) database and evaluated the reporting odds ratios (RORs) of tardive dyskinesia (TD) and other extrapyramidal symptoms (EPS) associated with antipsychotics. SGAs were evaluated both as a whole class and as subgroups based on their primary pharmacological mode of action. From 1 April 2011 to 31 March 2020, 1 197 065 ADRs, including 760 TD and 6059 EPS cases, were identified for this study. By calculating RORs, risk signals were detected with both FGAs and SGAs for TD and EPS compared with non‐antipsychotics, with an ROR (95% confidence interval (CI)) of 153.9 (125.64–188.34) with FGAs for TD and 95.3 (80.61–112.65) with SGAs total for TD. No risk signals were detected for SGAs total data or any SGA subgroups versus FGAs. The ROR (95% CI) with SGAs total versus FGAs for TD was 0.62 (0.51–0.75), for dyskinesia: 0.55 (0.42–0.72), and for parkinsonism: 0.43 (0.35–0.52), showing that SGAs were associated with lower reporting frequency versus FGAs, but not for akathisia and dystonia. In conclusion, both FGAs and SGAs were associated with risks for TD and EPS compared with non‐antipsychotics in the Japanese population, and SGAs total or all SGA subgroups showed no risk signals compared with FGAs.

## Abstract 2

From April 1, 2011 to March 31, 2020, 1 197 065 adverse drug reactions, including 760 TD and 6059 EPS cases, were identified for this study. By calculating reporting odds ratios, both First‐ and second‐generation antipsychotics (FGAs and SGAs) were associated with risks for tardive dyskinesia and extrapyramidal symptoms compared with non‐antipsychotics in the Japanese population, and SGAs total or all SGA subgroups showed no risk signals compared with FGAs.

## Graphical abstract

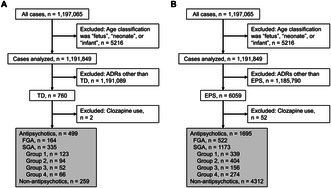

## 1 Introduction

Antipsychotics are used not only for the treatment of schizophrenia but also for other disorders, such as mood disorders and developmental disorders, leading to their widespread use compared with several decades ago [1, 2]. Antipsychotics improve symptoms mainly by blocking dopamine receptors and commonly induce various involuntary movement‐related adverse drug reactions (ADRs), such as extrapyramidal symptoms (EPS), including tremor, dyskinesia, dystonia, akathisia, parkinsonism, and tardive dyskinesia (TD) [3, 4, 5]. These ADRs may occur acutely after initiation of antipsychotic treatment or after long‐term use. The physical and psychological burden of such ADRs may negatively impact patients' quality of life and activities of daily living [6, 7, 8], possibly resulting in discontinuation of treatment or being disabled. Some risk and predictive factors for TD have been identified, such as older age, female sex, ethnicity, antipsychotic type/dose, concomitant use of other antipsychotics or anticholinergic agents, and early‐occurring EPS [9, 10, 11, 12].

First‐generation antipsychotics (FGAs) were developed in the 1950s. In pursuit of improved efficacy and fewer ADRs, many new drugs were approved in the 1980s, known as second‐generation antipsychotics (SGAs) [3, 8]. Both FGAs and SGAs are associated with TD and EPS, although SGAs are considered to have lower risks of these ADRs compared with FGAs [7, 13, 14, 15, 16]. However, the prevalence in the Japanese population, especially for TD, remains unclear. Assessing the reporting frequency of ADRs in an ADR database may identify potential risk signals for antipsychotic‐induced TD and EPS.

The Japanese Adverse Drug Event Report (JADER) database is published by the Pharmaceuticals and Medical Devices Agency [17]. The JADER collects spontaneous ADR information reported by patients and health care professionals, including physicians, pharmacists, and nurses. The database contains demographic information, including sex and age in subcategories, and ADRs, using terms found in the medical dictionary for regulatory activities/Japanese terminology (MedDRA/J). Drugs are reported with one of three categories defining a suspected association with the ADR: (1) suspected drug, indicating the drugs that were suspected to have caused the ADR; (2) interactions, indicating drugs that were suspected to have interacted with the suspected drug for the ADR; and (3) concomitant drug, indicating drugs that were taken concomitantly at the onset of the ADR.

Previously, we performed an analysis using the JADER database and demonstrated that TD and EPS were reported not only in patients with schizophrenia but also in those with mood disorders, such as bipolar disorder and major depressive disorder [18]; however, the risks of TD and EPS were not estimated. In this study, we sought to identify potential risk signals for antipsychotics‐induced TD and EPS by evaluating reporting odds ratios (RORs) between antipsychotics and non‐antipsychotics using the JADER database. We also assessed the RORs of TD and EPS by each class of SGAs versus FGAs.

## 2 Methods

### 2.1 Ethics Statement

This study used the JADER spontaneous reporting system database [17, 18]. This is a publicly accessible database for ADRs, and all information is anonymized. Therefore, review and approval from the institutional review board and informed consent were not required to perform this study.

### 2.2 Data Collection

All available ADR reports registered from 1 April 2011 to 31 March 2020 were obtained from the JADER database [17]. ADRs were defined by the reporting persons in accordance with the Diagnostic and Statistical Manual of Mental Disorders, 5th Edition, Text Revision (DSM‐5‐TR), and were collected as reported. The MedDRA/J at the time of this study was ver. 25.1. Multiple cases were treated as one case if all three of the following were identical: identification number, ADR, and generic name of the suspected drug. Cases were excluded in which the relationships between the drug and the ADR were classified as concomitant drug or drug–drug interactions, and the ADRs that were nonspecific and generally known as antipsychotic‐or injection‐related, such as long QT syndrome, anaphylaxis, and injection site reactions. After exclusion of ADR cases with an age category of “fetus,” “neonate,” or “infant,” ADR cases with TD or EPS (i.e., akathisia, dyskinesia, dystonia, parkinsonism, parkinsonian gait, bradykinesia, dysarthria, anarthria, cogwheel rigidity, tremor, akinesia, and hypersalivation) were selected. Furthermore, cases in which clozapine was used were excluded from the analysis. This medication is generally prescribed for treatment‐resistant schizophrenia after symptoms fail to improve with at least two other antipsychotics. ADRs reported for clozapine may have been influenced by prior use of other antipsychotics or their interactions, potentially confounding the analysis. The patients' demographic characteristics data were summarized for the remaining cases.

For the ROR analyses, multiple cases, as defined earlier, were also treated as one case. In addition to the previously noted exclusions, cases that could not be classified by age (< 60 or ≥ 60 years) or sex were excluded.

### 2.3 Antipsychotics

Reported antipsychotics were classified into two groups based on their generation: FGAs or SGAs. SGAs were further classified into groups 1–4 based on the pharmacology domain and mode of action proposed in the Neuroscience‐based Nomenclature (NbN) [19]: Group 1 includes antagonists of dopamine and serotonin receptors; group 2 includes antagonists of dopamine, serotonin, and norepinephrine receptors; group 3 includes multimodal antipsychotics that act on dopamine, serotonin, and norepinephrine receptors; and group 4 includes partial agonists and antagonists that act on dopamine and serotonin receptors. All drugs analyzed and their classifications are summarized in Table S1.

### 2.4 Statistical Analysis

For each reported case with TD or EPS, the following demographic information was collected and summarized in each category of antipsychotics: sex, age category, and concomitant use of FGAs, SGAs, anticholinergics, and lithium.

To detect risk signals, the disproportionality of ADR reporting frequency was analyzed using RORs [20]. The ROR method is often used to detect signals in data obtained from spontaneous reporting databases and is calculated using a two‐by‐two contingency table (Table S2). For the ROR analysis of each involuntary movement‐related ADR, one ADR was defined as the ADR of interest, and all other TD/EPS cases were excluded from the further analyses, leaving the ADR of interest and non‐TD/EPS ADR cases. The cases were classified first into two groups: ADR of interest or non‐TD/EPS ADR cases. Both groups were then classified into two groups by suspected drugs: antipsychotics or non‐antipsychotics. The antipsychotics groups were further classified into two groups by FGAs or SGAs. Finally, cases with SGAs were further classified into SGA groups 1–4.

The ROR was first calculated for antipsychotics (i.e., drug of interest) versus non‐antipsychotics as the reference (i.e., other drugs), using an ADR (i.e., ADR of interest) versus all non‐TD/EPS ADRs as the reference (i.e., other ADRs). For the second analysis, the ROR was calculated for SGAs (SGAs total or each SGA subgroup) as the drug of interest versus FGAs as the reference, using an ADR (i.e., ADR of interest) versus all non‐TD/EPS ADRs as the reference (i.e., other ADRs) (Table S2). Using Fisher's exact test, 95% confidence intervals (CIs) and *p* values were calculated for each ROR. It was considered that a significant risk signal was detected in the drug of interest when the lower limit (LL) of the 95% CI of the ROR exceeded 1.0, and in the reference drug when the upper limit (UL) of the 95% CI of the ROR did not reach 1.0. In addition to the crude ROR described above, adjusted RORs were calculated using a logistic regression model that included the following variables: age (< 60 years or ≥ 60 years), sex, reported year (2011–2015 or 2016–2020), concomitant use of FGAs (for SGA cases), concomitant use of SGAs (for FGA cases), concomitant use of anticholinergics, and concomitant use of lithium. For all statistical analyses, R Version 4.3.2 (www.r‐project.org, R Foundation for Statistical Computing, Vienna, Austria) was used.

## 3 Results

### 3.1 Characteristics of the Reported Cases

**FIGURE 1:**
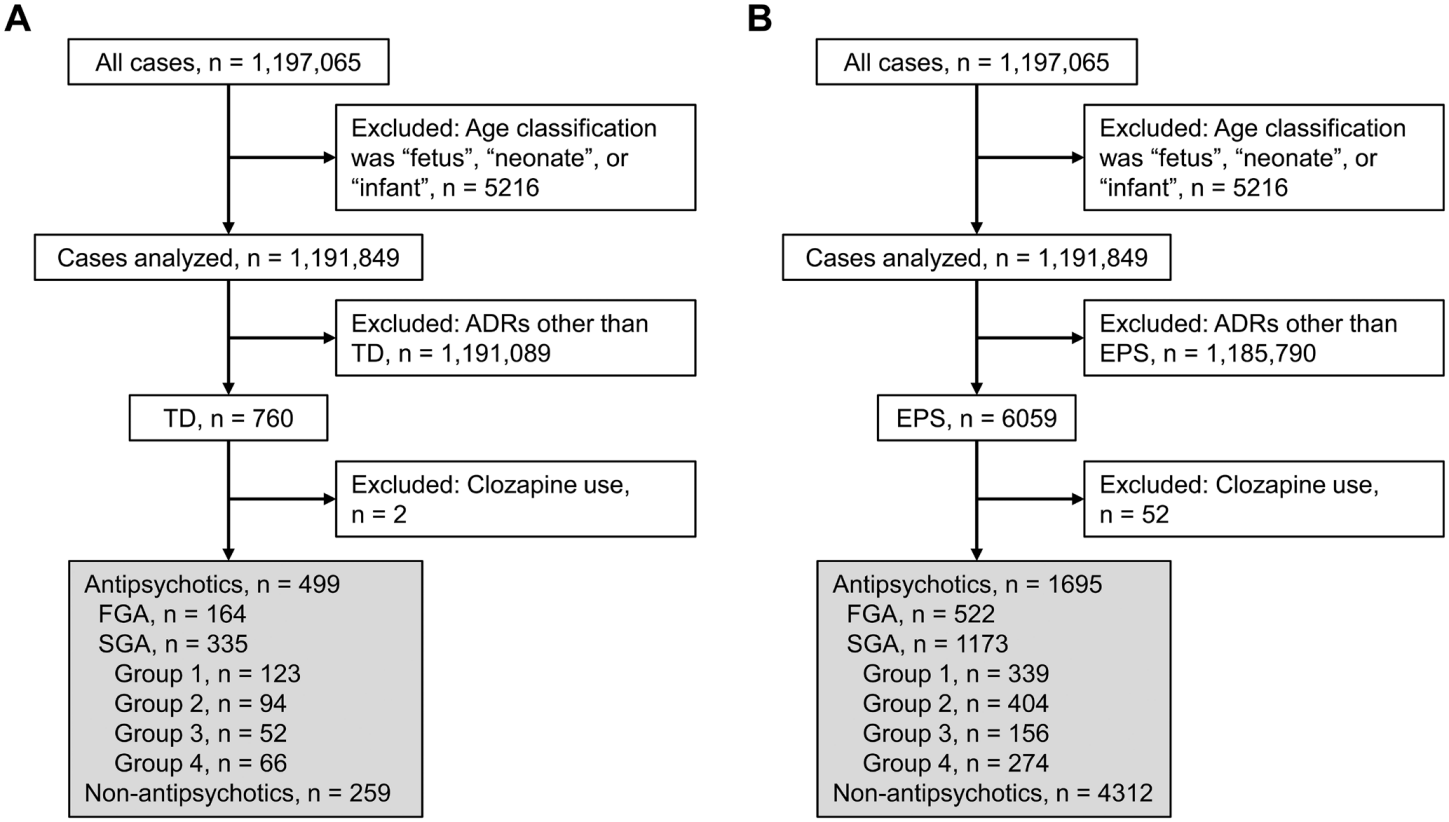
Flow diagram depicting the case selection process utilized in the demographic analysis. Panel A illustrates the selection of patients diagnosed with TD, while Panel B depicts the selection of patients exhibiting EPS. Cases enclosed within the gray boxes represent those included in the demographic analysis dataset. ADRs, adverse drug reactions; EPS, extrapyramidal symptoms; FGAs, first‐generation antipsychotics; SGAs, second‐generation antipsychotics; TD, tardive dyskinesia.

From all available ADR reports registered during the study period, multiplicated cases, cases associated with concomitant use and/or drug–drug interactions, and cases with ADRs generally known as antipsychotics‐induced were excluded. Data for 1 197 065 ADRs were used for this study. ADR cases with the age categories of “fetus” “neonate” or “infant” were further excluded. Among these cases (*n* = 1 191 849), 760 with TD and 6059 with EPS were identified (Figure 1). These cases were classified on the basis of the suspected drugs: For TD, suspected drugs were antipsychotics in 499 cases after exclusion of 2 clozapine cases (164 cases associated with FGAs and 335 cases associated with SGAs), and non‐antipsychotics in 259 cases (Figure 1A); for EPS, suspected drugs were antipsychotics in 1695 cases after exclusion of 52 clozapine cases (522 cases associated with FGAs and 1173 cases associated with SGAs), and non‐antipsychotics in 4312 cases (Figure 1B). Non‐antipsychotics frequently reported in association with these ADRs are summarized in Table S3.

The patients' characteristics for TD and EPS cases are summarized in Tables 1 and 2, respectively. The proportions of male patients among the TD cases were 57.93% (95/164 cases) for FGAs and 54.93% (184/335 cases) for SGAs. The proportions of male patients among the EPS cases were 44.64% (233/522 cases) for FGAs and 46.04% (540/1173 cases) for SGAs. TD cases with patients aged < 60 years comprised 105/164 cases (64.02%) for FGAs and 266/335 cases (79.40%) for SGAs, while cases with patients aged ≥ 60 years comprised 50/164 cases (30.49%) for FGAs and 57/335 cases (17.01%) for SGAs. EPS patients aged < 60 years comprised 264/522 cases (50.57%) for FGAs and 747/1173 cases (63.68%) for SGAs, while patients aged ≥ 60 years comprised 221/522 cases (42.34%) for FGAs and 335/1173 cases (28.56%) for SGAs.

**TABLE 1:** Characteristics of patients reported for tardive dyskinesia.

	FGAs	SGAs					Non‐antipsychotics
		Total	Group 1	Group 2	Group 3	Group 4	
*n*	164	335	123	94	52	66	259
Sex, *n* (%)							
Male	95 (57.93)	184 (54.93)	73 (59.35)	56 (59.57)	30 (57.69)	25 (37.88)	180 (69.50)
Female	68 (41.46)	150 (44.78)	49 (39.84)	38 (40.43)	22 (42.31)	41 (62.12)	79 (30.50)
Unknown	1 (0.61)	1 (0.30)	1 (0.81)	0 (0.00)	0 (0.00)	0 (0.00)	0 (0.00)
Age, years, *n* (%)							
< 20	2 (1.22)	11 (3.28)	0 (0.00)	5 (5.32)	2 (3.85)	4 (6.06)	3 (1.16)
20–39	29 (17.68)	98 (29.25)	41 (33.33)	36 (38.30)	9 (17.31)	12 (18.18)	35 (13.51)
40–59	74 (45.12)	157 (46.87)	58 (47.15)	33 (35.11)	32 (61.54)	34 (51.52)	178 (68.73)
60–79	46 (28.05)	49 (14.63)	17 (13.82)	12 (12.77)	6 (11.54)	14 (21.21)	40 (15.44)
≥ 80	3 (1.83)	8 (2.39)	2 (1.63)	4 (4.26)	2 (3.85)	0 (0.00)	2 (0.77)
Unknown	10 (6.10)	12 (3.58)	5 (4.07)	4 (4.26)	1 (1.92)	2 (3.03)	1 (0.39)
< 60 years	105 (64.02)	266 (79.40)	99 (80.49)	74 (78.72)	43 (82.69)	50 (75.76)	216 (83.4)
≥ 60 years	50 (30.49)	57 (17.01)	19 (15.45)	16 (17.02)	8 (15.38)	14 (21.21)	43 (16.6)
Unknown	9 (5.49)	12 (3.58)	5 (4.07)	4 (4.26)	1 (1.92)	2 (3.03)	0 (0.00)
Concomitant drug use, *n* (%)							
FGAs	0 (0.00)	169 (50.45)	71 (57.72)	32 (34.04)	39 (75.00)	27 (40.91)	182 (70.27)
SGAs	100 (60.98)	0 (0.00)	0 (0.00)	0 (0.00)	0 (0.00)	0 (0.00)	203 (78.38)
Anticholinergics	65 (39.63)	149 (44.48)	55 (44.72)	36 (38.30)	26 (50.00)	32 (48.48)	112 (43.24)
Lithium	10 (6.10)	28 (8.36)	13 (10.57)	8 (8.51)	4 (7.69)	3 (4.55)	13 (5.02)
Reported year, *n* (%)							
2011–2015	61 (37.20)	113 (33.73)	40 (32.52)	36 (38.30)	25 (48.08)	12 (18.18)	61 (23.55)
2016–2020	103 (62.80)	222 (66.27)	83 (67.48)	58 (61.70)	27 (51.92)	54 (81.82)	198 (76.45)

*Note:* When all three of the following—patient identification number, adverse drug reaction, and generic name of the suspected drug—were identical, the case was considered a single case, and duplicates were removed. Otherwise, a true single case may have been counted multiple times.

Abbreviations: FGAs, first‐generation antipsychotics; SGAs, second‐generation antipsychotics.

**TABLE 2:** Characteristics of patients reported for extrapyramidal symptoms.

	FGAs	SGAs					Non‐antipsychotics
		Total	Group 1	Group 2	Group 3	Group 4	
*n*	522	1173	339	404	156	274	4312
Sex, *n* (%)							
Male	233 (44.64)	540 (46.04)	158 (46.61)	184 (45.54)	71 (45.51)	127 (46.35)	2030 (47.08)
Female	286 (54.79)	624 (53.20)	178 (52.51)	215 (53.22)	84 (53.85)	147 (53.65)	2212 (51.30)
Unknown	3 (0.57)	9 (0.77)	3 (0.88)	5 (1.24)	1 (0.64)	0 (0.00)	70 (1.62)
Age, years, *n* (%)							
< 20	23 (4.41)	54 (4.60)	9 (2.65)	31 (7.67)	3 (1.92)	11 (4.01)	404 (9.37)
20–39	67 (12.84)	254 (21.65)	60 (17.70)	101 (25.00)	30 (19.23)	63 (22.99)	451 (10.46)
40–59	174 (33.33)	439 (37.43)	156 (46.02)	127 (31.44)	59 (37.82)	97 (35.40)	850 (19.71)
60–79	145 (27.78)	271 (23.10)	77 (22.71)	92 (22.77)	36 (23.08)	66 (24.09)	1532 (35.53)
≥ 80	73 (13.98)	59 (5.03)	9 (2.65)	20 (4.95)	19 (12.18)	11 (4.01)	732 (16.98)
Unknown	40 (7.66)	96 (8.18)	28 (8.26)	33 (8.17)	9 (5.77)	26 (9.49)	343 (7.95)
< 60	264 (50.57)	747 (63.68)	225 (66.37)	259 (64.11)	92 (58.97)	171 (62.41)	1712 (39.70)
≥ 60	221 (42.34)	335 (28.56)	88 (25.96)	113 (27.97)	57 (36.54)	77 (28.10)	2279 (52.85)
Unknown	37 (7.09)	91 (7.76)	26 (7.67)	32 (7.92)	7 (4.49)	26 (9.49)	321 (7.44)
Concomitant drug use, *n* (%)							
FGAs	0 (0.00)	377 (32.14)	133 (39.23)	105 (25.99)	72 (46.15)	67 (24.45)	598 (13.87)
SGAs	220 (42.15)	0 (0.00)	0 (0.00)	0 (0.00)	0 (0.00)	0 (0.00)	880 (20.41)
Anticholinergics	123 (23.56)	373 (31.80)	115 (33.92)	127 (31.44)	64 (41.03)	67 (24.45)	446 (10.34)
Lithium	15 (2.87)	97 (8.27)	29 (8.55)	29 (7.18)	18 (11.54)	21 (7.66)	171 (3.97)
Reported year, *n* (%)							
2011–2015	258 (49.43)	532 (45.35)	133 (39.23)	223 (55.20)	92 (58.97)	84 (30.66)	2189 (50.77)
2016–2020	264 (50.57)	641 (54.65)	206 (60.77)	181 (44.80)	64 (41.03)	190 (69.34)	2123 (49.23)

*Note:* When all three of the following—patient identification number, adverse drug reaction, and generic name of the suspected drug—were identical, the case was considered a single case, and duplicates were removed. Otherwise, a true single case may have been counted multiple times.

Abbreviations: FGAs: first‐generation antipsychotics; SGAs: second‐generation antipsychotics.

Concomitant SGA use was reported for TD in 100/164 cases (60.98%) and for EPS in 220/522 cases (42.15%) among patients with FGAs as the suspected drug. In comparison, concomitant FGA use was reported for TD in 169/335 cases (50.45%) and for EPS in 377/1173 cases (32.14%) among patients with SGAs as the suspected drug. Anticholinergic agents were used in cases reported for TD (FGAs: 65/164 cases, 39.63%; SGAs: 149/335 cases, 44.48%) and EPS (FGAs: 123/522 cases, 23.56%; SGAs: 373/1173 cases, 31.80%). Lithium was used in cases reported for TD (FGAs: 10/164 cases, 6.10%; SGAs: 28/335 cases, 8.36%) and EPS (FGAs: 15/522 cases, 2.87%; SGAs: 97/1173 cases, 8.27%).

### 3.2 Reporting Odds Ratios

**FIGURE 2:**
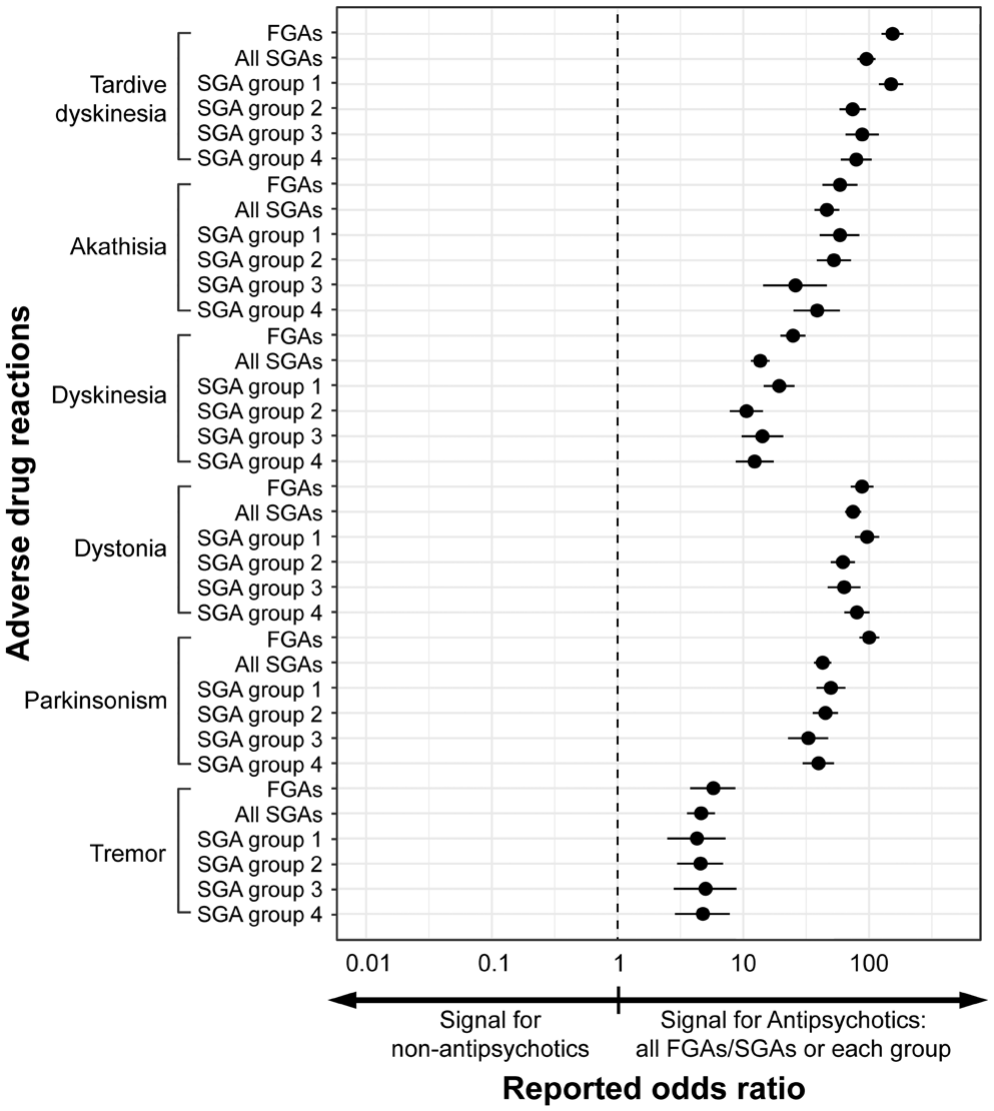
Forest plot illustrating the crude RORs for potential safety signals associated with each ADR across different antipsychotic categories compared to non‐antipsychotics. Crude RORs with 95% CIs are displayed on a logarithmic scale. RORs greater than 1 suggest a higher likelihood of reporting an ADR in association with antipsychotic exposure relative to non‐antipsychotic agents. The remaining ADRs analyzed are summarized in Figure S1. ADR, adverse drug reaction; CIs, confidence intervals; FGAs, first‐generation antipsychotics; RORs, reporting odds ratios; SGAs, second‐generation antipsychotics.

To detect risk signals for an ADR, we calculated the RORs by setting the ADR as the ADR of interest and removing all other TD/EPS ADRs from the analysis. The number of cases for each ADR used to calculate RORs is summarized in Table S4. Crude RORs with 95% CIs for the risk of each ADR by antipsychotics (FGAs, SGAs total, and SGA groups 1–4) versus non‐antipsychotics are summarized in Figures 2 and S1, and Table 3. For most TD/EPS ADRs, the LLs of the 95% CIs of the RORs were higher than 1, with the highest ROR (95% CI) of 153.9 (125.64–188.34) for TD associated with FGAs. These findings indicate that there were risk signals for an association between antipsychotics and involuntary movement‐related ADRs. Signals were detected with both FGAs and SGAs total versus non‐antipsychotics for all evaluated ADRs except Parkinsonian gait with FGAs (Figures 2 and S1), as well as all SGA subgroups for TD, akathisia, dyskinesia, dystonia, parkinsonism, tremor, and hypersalivation.

**TABLE 3:** Crude RORs and adjusted RORs in each antipsychotics category versus non‐antipsychotics.

ADR of interest	Crude ROR	95% CI	*p*	Adjusted ROR	95% CI	*p*
Antipsychotics category						
Tardive dyskinesia						
FGAs	153.9	125.64–188.34	< 0.001	108.21	76.23–153.59	< 0.001
SGAs total	95.3	80.61–112.65	< 0.001	136.6	99.98–186.62	< 0.001
SGA group 1	149.73	119.50–187.48	< 0.001	134.02	91.73–195.82	< 0.001
SGA group 2	74.08	58.10–94.50	< 0.001	80.49	54.74–118.34	< 0.001
SGA group 3	88.31	64.96–119.49	< 0.001	73.57	46.66–116.00	< 0.001
SGA group 4	79.14	59.66–104.89	< 0.001	76.55	50.61–115.78	< 0.001
Akathisia (*n*)						
FGAs	58.77	42.47–81.04	< 0.001	20.93	13.96–31.39	< 0.001
SGAs total	46.15	36.77–57.88	< 0.001	44.5	33.81–58.58	< 0.001
SGA group 1	58.74	40.49–83.75	< 0.001	51.01	33.71–77.18	< 0.001
SGA group 2	52.52	38.32–71.87	< 0.001	49.53	34.30–71.54	< 0.001
SGA group 3	26	14.35–46.16	< 0.001	22.17	11.92–41.24	< 0.001
SGA group 4	38.68	25.00–58.79	< 0.001	33.11	20.82–52.68	< 0.001
Dyskinesia (*n*)						
FGAs	24.86	19.70–31.19	< 0.001	7.84	5.90–10.41	< 0.001
SGAs total	13.64	11.48–16.17	< 0.001	6.42	5.16–7.99	< 0.001
SGA group 1	19.31	14.50–25.58	< 0.001	7.2	5.16–10.04	< 0.001
SGA group 2	10.6	7.80–14.33	< 0.001	4.09	2.89–5.80	< 0.001
SGA group 3	14.19	9.68–20.84	< 0.001	6.75	4.47–10.19	< 0.001
SGA group 4	12.3	8.69–17.46	< 0.001	5.97	4.09–8.71	< 0.001
Dystonia (*n*)						
FGAs	87.92	71.36–108.30	< 0.001	23.59	17.29–32.20	< 0.001
SGAs total	74.55	64.28–86.43	< 0.001	76.39	60.57–96.36	< 0.001
SGA group 1	96.45	77.16–120.19	< 0.001	62.31	45.48–85.37	< 0.001
SGA group 2	61.99	49.59–77.19	< 0.001	52.59	38.92–71.06	< 0.001
SGA group 3	63.37	46.79–85.48	< 0.001	45.79	31.18–67.24	< 0.001
SGA group 4	80.06	63.46–101.09	< 0.001	65.49	47.95–89.46	< 0.001
Parkinsonism (*n*)						
FGAs	100.33	83.72–120.47	< 0.001	113.63	90.54–142.61	< 0.001
SGAs total	42.77	36.47–50.10	< 0.001	51.58	42.40–62.74	< 0.001
SGA group 1	49.75	38.12–64.85	< 0.001	45.19	32.98–61.92	< 0.001
SGA group 2	44.98	35.67–56.57	< 0.001	43.25	32.78–57.06	< 0.001
SGA group 3	32.92	22.64–47.22	< 0.001	25.25	16.95–37.63	< 0.001
SGA group 4	39.67	29.61–52.63	< 0.001	40.49	29.22–56.10	< 0.001
Parkinsonian gait (*n*)						
FGAs	14.38	0.70–89.65	0.07	6.88	0.68–69.23	0.10
SGAs total	21.23	7.54–58.48	< 0.001	36.15	11.96–109.26	< 0.001
SGA group 1	18.26	0.89–113.85	0.06	23.87	2.61–218.31	0.005
SGA group 2	0	NA–NA	> 0.99	0	0.00–Inf	0.99
SGA group 3	0	NA–NA	> 0.99	0	0.00–Inf	> 0.99
SGA group 4	71.17	22.10–203.64	< 0.001	121.03	36.08–405.98	< 0.001
Bradykinesia (*n*)						
FGAs	18.49	3.14–70.29	0.006	3.18	0.66–15.38	0.15
SGAs total	46.4	24.36–87.50	< 0.001	69.2	33.13–144.53	< 0.001
SGA group 1	58.69	21.58–151.98	< 0.001	95.99	32.85–280.43	< 0.001
SGA group 2	45.68	18.47–108.32	< 0.001	65.04	23.67–178.73	< 0.001
SGA group 3	16.02	0.79–91.23	0.06	19.5	2.48–153.20	0.005
SGA group 4	57.19	21.03–148.10	< 0.001	77.21	26.26–227.04	< 0.001
Dysarthria (*n*)						
FGAs	5.18	1.75–13.77	0.008	4.37	1.50–12.74	0.007
SGAs total	4.59	2.53–8.13	< 0.001	3.3	1.68–6.47	< 0.001
SGA group 1	6.57	2.22–17.50	0.004	3.75	1.26–11.14	0.02
SGA group 2	3.2	0.86–9.67	0.07	1.95	0.57–6.61	0.28
SGA group 3	4.48	0.79–16.82	0.08	3.03	0.72–12.77	0.13
SGA group 4	4.8	1.30–14.53	0.03	3.31	1.00–10.95	0.05
Anarthria (*n*)						
FGAs	4.46	2.36–8.31	< 0.001	3.34	1.70–6.54	< 0.001
SGAs total	3.43	2.26–5.09	< 0.001	3.29	2.14–5.05	< 0.001
SGA group 1	1.7	0.46–5.05	0.26	1.46	0.46–4.66	0.52
SGA group 2	5.15	2.97–8.73	< 0.001	4.99	2.83–8.79	< 0.001
SGA group 3	3.87	1.52–9.25	0.01	3.43	1.40–8.42	0.007
SGA group 4	2.21	0.75–5.76	0.11	1.95	0.71–5.31	0.19
Cogwheel rigidity (*n*)						
FGAs	0	NA–NA	> 0.99	0	0.00–Inf	> 0.99
SGAs total	0	NA–NA	> 0.99	0	0.00–Inf	> 0.99
SGA group 1	0	NA–NA	> 0.99	0	0.00–Inf	> 0.99
SGA group 2	0	NA–NA	> 0.99	0	0.00–Inf	> 0.99
SGA group 3	0	NA–NA	> 0.99	0	0.00–Inf	> 0.99
SGA group 4	0	NA–NA	> 0.99	0	0.00–Inf	> 0.99
Tremor (*n*)						
FGAs	5.78	3.77–8.64	< 0.001	3.39	2.18–5.28	< 0.001
SGAs total	4.62	3.57–5.96	< 0.001	4.36	3.31–5.74	< 0.001
SGA group 1	4.28	2.48–7.23	< 0.001	3.63	2.09–6.30	< 0.001
SGA group 2	4.57	2.97–6.91	< 0.001	3.96	2.55–6.13	< 0.001
SGA group 3	5.01	2.79–8.81	< 0.001	4.45	2.49–7.95	< 0.001
SGA group 4	4.77	2.86–7.80	< 0.001	4.24	2.55–7.06	< 0.001
Akinesia (*n*)						
FGAs	21.57	9.84–46.59	< 0.001	8.02	3.06–21.03	< 0.001
SGAs total	21.83	13.65–34.30	< 0.001	15.29	8.50–27.50	< 0.001
SGA group 1	39.12	19.76–74.80	< 0.001	18.36	7.84–43.00	< 0.001
SGA group 2	25.38	12.82–48.49	< 0.001	9.86	4.22–23.08	< 0.001
SGA group 3	5.34	0.27–30.09	0.17	1.79	0.23–13.87	0.58
SGA group 4	11.44	3.06–34.10	0.003	4.33	1.21–15.47	0.02
Hypersalivation (*n*)						
FGAs	23.01	10.30–46.47	< 0.001	5.4	2.26–12.93	< 0.001
SGAs total	35.66	24.34–52.06	< 0.001	25.47	15.98–40.59	< 0.001
SGA group 1	43.82	23.66–79.83	< 0.001	18.2	8.57–38.67	< 0.001
SGA group 2	42.64	24.98–70.89	< 0.001	25.13	13.39–47.17	< 0.001
SGA group 3	19.93	6.67–52.64	< 0.001	9.6	3.19–28.94	< 0.001
SGA group 4	28.47	12.74–57.52	< 0.001	14	6.09–32.20	< 0.001

Abbreviations: ADR, adverse drug reaction; CI, confidence interval; FGAs, first‐generation antipsychotics; Inf, infinite; NA, not applicable; ROR, reporting odds ratio; SGAs, second‐generation antipsychotics.

**FIGURE 3:**
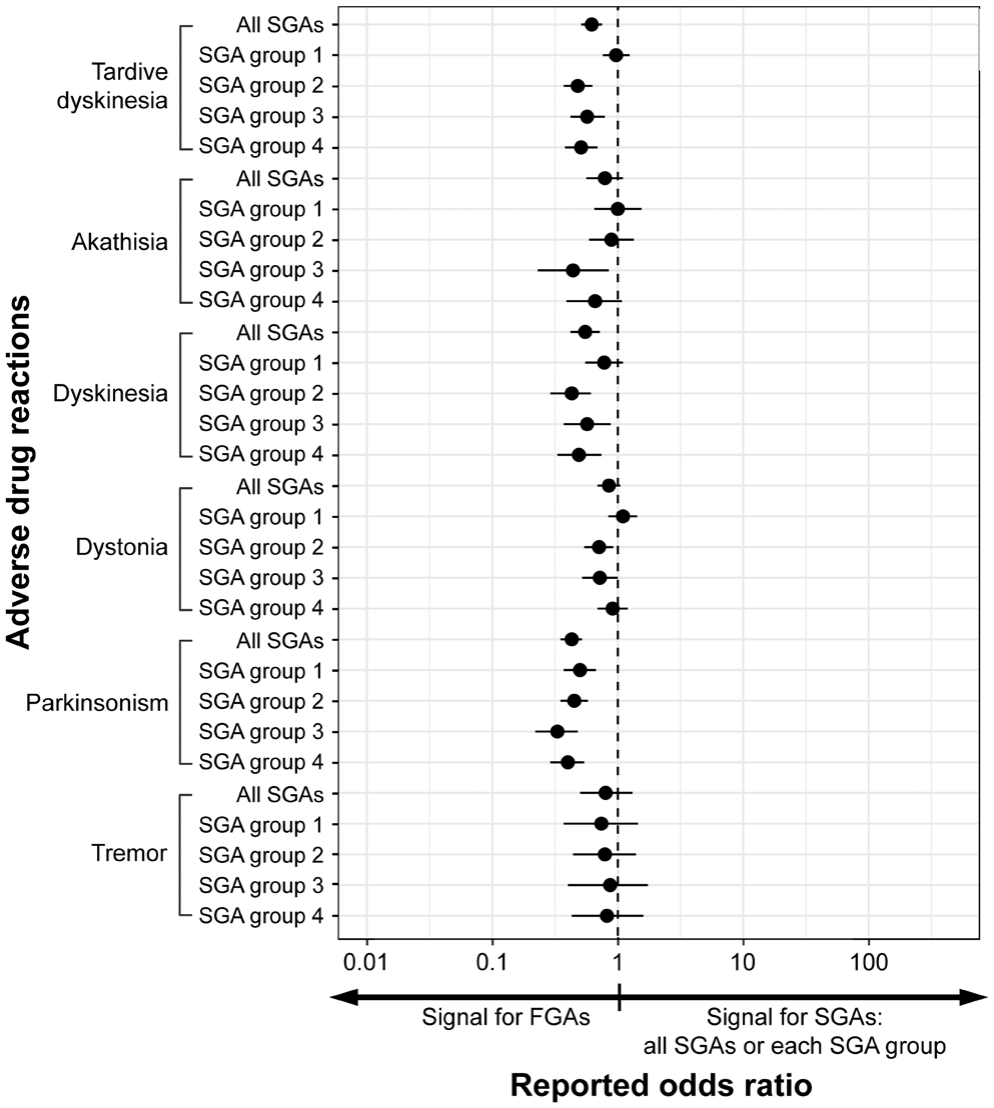
Forest plot depicting the crude RORs for potential safety signals related to each ADR across SGA categories compared to FGA. Crude RORs with 95% CIs are plotted on a logarithmic scale. RORs exceeding 1 indicate an elevated signal for ADR reporting associated with SGAs relative to FGAs. The full list of other analyzed ADRs is presented in Figure S2. ADR, adverse drug reaction; CIs, confidence intervals; FGAs, first‐generation antipsychotics; RORs, reporting odds ratios; SGAs, second‐generation antipsychotics.

The analyses of SGAs total and for each SGA group versus FGAs detected no signals in any SGA group for all evaluated ADRs (Figures 3 and S2). The ULs of the 95% CIs when comparing SGAs total versus FGAs were less than 1 for TD, dyskinesia, and parkinsonism, showing that signals were found with FGAs versus SGAs (Figures 3 and S2, and Table 4).

**TABLE 4:** Crude RORs and adjusted RORs in each SGA category versus FGAs.

ADR of interest	Crude ROR	95% CI	*p*	Adjusted ROR	95% CI	*p*
Antipsychotics category						
Tardive dyskinesia						
SGAs total	0.62	0.51–0.75	< 0.001	0.5	0.37–0.68	< 0.001
SGA group 1	0.97	0.76–1.24	0.85	0.72	0.49–1.07	0.10
SGA group 2	0.48	0.37–0.63	< 0.001	0.54	0.38–0.78	0.001
SGA group 3	0.57	0.42–0.79	< 0.001	0.23	0.12–0.43	< 0.001
SGA group 4	0.51	0.38–0.69	< 0.001	0.48	0.32–0.72	< 0.001
Akathisia (*n*)						
SGAs total	0.79	0.56–1.10	0.17	0.78	0.48–1.27	0.32
SGA group 1	1	0.65–1.55	> 0.99	1.12	0.62–2.02	0.71
SGA group 2	0.89	0.59–1.34	0.61	0.95	0.55–1.64	0.85
SGA group 3	0.44	0.23–0.85	0.009	0.27	0.10–0.71	0.008
SGA group 4	0.66	0.39–1.08	0.10	0.78	0.42–1.44	0.43
Dyskinesia (*n*)						
SGAs total	0.55	0.42–0.72	< 0.001	0.41	0.28–0.61	< 0.001
SGA group 1	0.78	0.55–1.10	0.16	0.49	0.28–0.85	0.01
SGA group 2	0.43	0.29–0.61	< 0.001	0.36	0.21–0.60	< 0.001
SGA group 3	0.57	0.37–0.88	0.01	0.42	0.22–0.77	0.006
SGA group 4	0.49	0.33–0.74	< 0.001	0.41	0.24–0.72	0.002
Dystonia (*n*)						
SGAs total	0.85	0.69–1.05	0.12	0.82	0.60–1.13	0.23
SGA group 1	1.1	0.84–1.43	0.50	0.89	0.59–1.33	0.57
SGA group 2	0.71	0.54–0.92	0.009	0.91	0.63–1.31	0.62
SGA group 3	0.72	0.52–1.00	0.06	0.43	0.25–0.76	0.003
SGA group 4	0.91	0.69–1.20	0.53	0.87	0.59–1.28	0.48
Parkinsonism (*n*)						
SGAs total	0.43	0.35–0.52	< 0.001	0.31	0.25–0.39	< 0.001
SGA group 1	0.5	0.37–0.67	< 0.001	0.4	0.29–0.56	< 0.001
SGA group 2	0.45	0.35–0.58	< 0.001	0.34	0.25–0.46	< 0.001
SGA group 3	0.33	0.22–0.48	< 0.001	0.18	0.11–0.28	< 0.001
SGA group 4	0.4	0.29–0.54	< 0.001	0.31	0.22–0.45	< 0.001
Parkinsonian gait (*n*)						
SGAs total	1.48	0.21–34.40	> 0.99	1.32	0.15–11.41	0.80
SGA group 1	1.27	0.03–48.89	> 0.99	1.8	0.11–29.24	0.68
SGA group 2	0	NA–NA	0.45	0	0.00–Inf	> 0.99
SGA group 3	0	NA–NA	> 0.99	0	0.00–Inf	> 0.99
SGA group 4	4.95	0.64–120.02	0.18	4.64	0.51–42.30	0.17
Bradykinesia (*n*)						
SGAs total	2.51	0.64–15.22	0.28	3.08	0.40–23.49	0.28
SGA group 1	3.17	0.66–22.53	0.25	5.39	0.61–47.42	0.13
SGA group 2	2.47	0.47–16.94	0.31	3.06	0.35–26.60	0.31
SGA group 3	0.87	0.03–11.07	> 0.99	0.74	0.04–12.72	0.84
SGA group 4	3.09	0.64–21.95	0.25	3.48	0.38–31.75	0.27
Dysarthria (*n*)						
SGAs total	0.89	0.29–2.98	0.77	0.61	0.19–1.93	0.40
SGA group 1	1.27	0.30–5.32	0.74	0.86	0.21–3.55	0.83
SGA group 2	0.62	0.12–2.83	0.71	0.41	0.09–1.85	0.24
SGA group 3	0.87	0.12–4.66	> 0.99	0.59	0.11–3.29	0.55
SGA group 4	0.93	0.18–4.26	> 0.99	0.5	0.11–2.32	0.38
Anarthria (*n*)						
SGAs total	0.77	0.37–1.71	0.43	0.81	0.35–1.89	0.62
SGA group 1	0.38	0.09–1.37	0.17	0.37	0.08–1.81	0.22
SGA group 2	1.15	0.49–2.70	0.84	1.28	0.50–3.23	0.61
SGA group 3	0.87	0.29–2.64	> 0.99	0.85	0.27–2.69	0.78
SGA group 4	0.49	0.14–1.67	0.29	0.63	0.18–2.19	0.47
Cogwheel rigidity (*n*)						
SGAs total	NA	NA–NA	> 0.99	1	0.00–Inf	> 0.99
SGA group 1	NA	NA–NA	> 0.99	1	0.00–Inf	> 0.99
SGA group 2	NA	NA–NA	> 0.99	1	0.00–Inf	> 0.99
SGA group 3	NA	NA–NA	> 0.99	1	0.00–Inf	> 0.99
SGA group 4	NA	NA–NA	> 0.99	1	0.00–Inf	> 0.99
Tremor (*n*)						
SGAs total	0.8	0.50–1.31	0.37	0.94	0.51–1.73	0.84
SGA group 1	0.74	0.37–1.45	0.42	0.86	0.37–1.98	0.72
SGA group 2	0.79	0.44–1.40	0.46	0.96	0.47–1.96	0.91
SGA group 3	0.87	0.40–1.74	0.73	0.89	0.39–2.04	0.78
SGA group 4	0.82	0.43–1.60	0.63	1.1	0.52–2.31	0.81
Akinesia (*n*)						
SGAs total	1.01	0.44–2.44	> 0.99	0.85	0.28–2.56	0.77
SGA group 1	1.81	0.68–5.21	0.23	1.86	0.55–6.29	0.32
SGA group 2	1.18	0.44–3.38	0.81	1	0.28–3.58	> 0.99
SGA group 3	0.25	0.01–1.74	0.27	0	0.00–Inf	> 0.99
SGA group 4	0.53	0.12–2.01	0.53	0.57	0.10–3.14	0.52
Hypersalivation (*n*)						
SGAs total	1.55	0.73–3.65	0.31	3.13	0.75–13.15	0.12
SGA group 1	1.9	0.75–4.81	0.18	2.86	0.59–13.86	0.19
SGA group 2	1.85	0.81–4.52	0.17	3.9	0.88–17.28	0.07
SGA group 3	0.87	0.24–2.95	> 0.99	1.52	0.25–9.27	0.65
SGA group 4	1.24	0.46–3.31	0.80	2.33	0.46–11.78	0.31

Abbreviations: ADR, adverse drug reaction; CI, confidence interval; FGAs, first‐generation antipsychotics; Inf, infinite; NA, not applicable; ROR, reporting odds ratio; SGAs, second‐generation antipsychotics.

Crude and adjusted RORs for the risk of each ADR by antipsychotics (FGAs, SGAs total, and SGA groups 1–4) versus non‐antipsychotics and FGAs versus SGAs total and SGA groups 1–4 showed overall similar trends in signal detection (Tables 3 and 4). For antipsychotics versus non‐antipsychotics, the crude RORs detected a risk signal with FGAs for bradykinesia (LL of the 95% CI = 3.14), but the signal was no longer detected after adjustment (LL of the 95% CI = 0.66). Notably, the crude ROR did not detect risk signals in SGA group 1 for Parkinsonian gait, but the adjusted ROR did (LL of the 95% CI by crude ROR = 0.89 and by adjusted ROR = 2.61). This was also true for SGA group 3 for bradykinesia (LL of the 95% CI by crude ROR = 0.79 and by adjusted ROR = 2.48) (Table 3). There were no such differences in signal detection between crude and adjusted RORs for other ADRs, including any major TD/ESP. For the comparison between SGAs total or each SGA group versus FGAs, the crude ROR detected a risk signal with FGAs versus SGA group 2 for dystonia (UL of the 95% CI by crude ROR = 0.92), but the signal was no longer detected after adjustment (UL of the 95% CI by crude ROR = 1.31). Notably, the crude ROR did not detect a risk signal with FGAs versus SGA group 1 for dyskinesia, but the adjusted ROR did (UL of the 95% CI by crude ROR = 1.10 and by adjusted ROR = 0.85). This was also true for FGAs versus SGA group 3 for dystonia (UL of the 95% CI by crude ROR = 1.00 and by adjusted ROR = 0.76) (Table 4).

## 4 Discussion

In this study, we evaluated the associations between antipsychotics and involuntary movement‐related ADRs, specifically TD and EPS, using data from the JADER database, a nationwide ADR reporting system in Japan. The ROR analysis revealed that both FGAs and SGAs are associated with risk signals for TD and EPS compared with non‐antipsychotic medications. These results are robust because signals for TD and EPS were detected consistently in both crude and adjusted RORs. Additionally, compared with FGAs, the SGAs total group exhibited lower risk signals for TD, dyskinesia, and parkinsonism, but not for akathisia, dystonia, and tremor. No signals with the SGAs total group or any SGA subgroups were detected versus the FGAs group. Regarding parkinsonism, the UL of the 95% CI for both crude and adjusted RORs did not exceed 1 for SGAs total or any SGA groups versus FGAs.

Our study, conducted in Japan, showed that both SGAs and FGAs were associated with increased risks for involuntary movement‐related ADRs compared with non‐antipsychotic medications, consistent with results in several previous overseas studies [3, 4, 5, 13]. It has been suggested that SGAs generally pose lower risks for inducing such ADRs compared with FGAs, particularly for TD [9, 13, 14, 15]. However, the difference in risk was less clear for EPS [21, 22]. Although approved SGAs exhibit different profiles from FGAs, such as non‐dopamine D2 receptor antagonism, it is not surprising that SGAs are still associated with risks for TD and EPS. Therefore, careful attention should be paid to these ADRs in clinical practice, even when SGAs are prescribed.

Different SGAs exhibit variable ADR profiles, including their propensity to induce TD and EPS. In this study, we defined the SGA groups based on the NbN nomenclature [19], a framework designed to provide more scientifically accurate and clinically relevant information compared with traditional classifications of psychiatric medications. For TD, both crude and adjusted ROR analyses indicated that FGAs exhibited risk signals compared with SGA subgroups 2, 3, and 4, but not with SGA group 1 (crude ROR for SGA group 1 vs. FGAs: 0.97, 95% CI: 0.76–1.24; adjusted ROR for SGA group 1 vs. FGAs: 0.72, 95% CI: 0.49–1.07). Similarly, there was no risk signal with FGAs versus SGA group 1 in the crude ROR for dyskinesia. In contrast, FGAs consistently showed risk signals for TD or dyskinesia versus SGA groups 2, 3, and 4 across all analyses.

While it is true that ROR values are not intended to provide accurate estimates of the relative risks of different medications, our findings are consistent with those in a recent head‐to‐head randomized controlled trial comparing paliperidone (SGA subgroup 2) and aripiprazole (SGA subgroup 4) as both oral and long‐acting injectable formulations among patients with early‐phase schizophrenia [23]. In that study, there were no clinically or statistically noticeable differences in the incidence of TD or dyskinesia between the two agent groups during the 19‐month observation period. With these considerations, our data support the idea that patients who receive dopamine and serotonin partial agonists (SGA subgroup 4) should be actively screened for TD or dyskinesia, as for those who receive other SGAs.

Regarding other EPS domains, FGAs exhibited risk signals for parkinsonism compared with the SGAs total group. However, FGAs showed risk signals versus SGA subgroup 3 (quetiapine) for akathisia and dystonia. The results were consistent with the findings from a network meta‐analysis of randomized controlled trials, which showed that quetiapine caused a relatively low frequency of EPS (e.g., incidence of akathisia and the use of anti‐parkinsonism medications to indicate extrapyramidal side effects) compared with other frequently used antipsychotic agents [24].

In the JADER database, the number of reported cases was distributed across all age groups, indicating that TD and EPS could be induced at any age. This finding is consistent with those of our previous analysis [18]. Patients under 60 years of age constitute a larger proportion of TD and EPS cases reported in the database, irrespective of the type of potential culprit medications. Although younger age is generally associated with a lower risk of TD [10], the increasing use of SGAs in the younger patient population and for a broader range of non‐psychotic indications could have led to the high number of TD cases. Clinicians should be mindful of this possibility and prescribe SGAs in a careful, evidence‐based manner whenever possible.

Anticholinergic agents were used concomitantly in approximately 40% of the patients with TD and 25% of those with EPS at the time of the ADR report, in our data analysis. The high proportion of anticholinergic use at the time of TD reporting deserves special attention. Early EPS is one of the strongest risk factors for TD [25]; therefore, proactive screening and detection for TD are important. The best clinical practice to prevent the development of TD involves adjusting the antipsychotic regimen. For example, when EPS develops, possible adjustments include either lowering the dosage or switching to another agent with a lower EPS propensity, according to patients' individual tolerability profiles, rather than simply adding anticholinergic agents to suppress EPS symptoms. Additionally, evidence suggests that anticholinergic agents may induce supersensitivity in dopamine receptors [26, 27], are ineffective for TD [28], or can aggravate or unmask TD symptoms [29]. Clinicians should be aware of the risks of EPS associated with the inappropriate, long‐term use of anticholinergic agents in patients taking any antipsychotics [29].

Currently, Vesicular Monoamine Transporter 2 (VMAT2) inhibitors such as Tetrabenazine, Valbenazine, and Deutetrabenazine are available for the treatment of TD [30, 31, 32]. These are important options to reduce the symptoms of TD for improvement of QOL.

## 5 Limitations

This study used data from JADER, a spontaneous reporting system, and we calculated RORs to detect a potential association between the drug of interest and the ADR of interest [20]. However, the primary evaluation method from such a database, ROR calculation, has several limitations. The database lacks information on prior treatments, including the type, duration, and dosage of antipsychotics used, making it unclear whether prior treatments have influenced ADR occurrence. Another missing piece of information in the database is the total number of patients administered antipsychotics, resulting from the lack of data on patients who did not experience ADRs. Due to this missing information, the “true” risks for TD or EPS could not be calculated. Moreover, the analysis did not account for cumulative or interacting effects of the drug of interest plus concomitant drugs, including other antipsychotics, despite many registered cases involving polypharmacy. Additionally, the absolute number of total antipsychotics prescribed cannot be determined. SGAs are newer drugs than FGAs; therefore, ADRs may have been reported more frequently for SGAs than those for FGAs because ADR reporting tends to be more frequent for newer drugs. Furthermore, information on antipsychotic dosage and the primary disease and its clinical severity was also either unknown or not accurate enough to allow for further analyses. Moreover, the causal relationship between the drug of interest and the reported ADR was determined subjectively by the reporting person and, therefore, not validated. Similarly, ADRs were collected as reported by patients or health care professionals without formal verification. For example, TD is differentiated from dyskinesia in the DSM‐5‐TR by the latency to onset (≥ 3 months), but the treatment duration was not reported. As such, this ROR method could detect only risk “signals” instead of the actual risks. Despite these limitations, the database contains a high volume of real‐world data, and the risk signals derived from these data provide valuable information.

## 6 Conclusions

Using ADR data from a Japanese spontaneous reporting system database, we demonstrated that antipsychotics were associated with more involuntary movement‐related ADRs, such as TD and other EPS, compared with non‐antipsychotic agents. On the basis of the RORs, risk signals were detected for both TD and EPS associated with FGAs and SGAs.

## Author Contributions

Y.S., H.H., C.L.C., and A.W. designed the study. Y.S. and H.H. interpreted the results and assisted with the writing of the manuscript. C.L.C. supervised the project. Y.S. and H.H. wrote the manuscript with support from C.L.C. All authors discussed the results and commented on the manuscript.

## Consent

The authors have nothing to report.

## Conflicts of Interest

All authors are employees of Johnson and Johnson, Japan.

## Supporting information


**Figure S1:** Forest Plot of the Crude Reporting Odds Ratios for Risk Signals for Reporting Each Adverse Drug Reaction for Each Antipsychotics Category versus Non‐Antipsychotics Crude RORs with 95% CIs are plotted on a log scale. Crude RORs greater than 1 indicate higher risk signals of reporting an ADR associated with antipsychotics versus non‐antipsychotics. ADR, adverse drug reaction; CIs, confidence intervals; FGAs, first‐generation antipsychotics; RORs, reporting odds ratios; SGAs, second‐generation antipsychotics.
**Figure S2:** Forest Plot of the Crude Reporting Odds Ratios for Risk Signals for Reporting Each Adverse Drug Reaction for Each Second‐Generation Antipsychotics Category versus First‐Generation Antipsychotics Crude RORs with 95% CIs are plotted on a log scale. Crude RORs greater than 1 indicate higher risk signals for reporting an ADR associated with SGAs versus FGAs. ADR, adverse drug reaction; CIs, confidence intervals; FGAs, first‐generation antipsychotics; RORs, reporting odds ratios; SGAs, second‐generation antipsychotics.
**Table S1:** Classification of antipsychotics.
**Table S2:** Two‐by‐two contingency table to calculate reporting odds ratios.
**Table S3:** Non‐antipsychotics reported in ≥ 5 cases for tardive dyskinesia or ≥ 10 cases for extrapyramidal symptoms in alphabetical order.
**Table S4:** Cases of adverse drug reactions of interest used to calculate reporting odds ratios using the two‐by‐two contingency table described in Table S2.

## Data Availability

The data that support the findings of this study are openly available in the JADER database at https://www.pmda.go.jp/safety/info‐services/drugs/adr‐info/suspected‐adr/0005.html (in Japanese). The datasets used for the statistical analyses that support the findings of this study are available from the corresponding author upon reasonable request.
