# Supplementary material for: Reporting Frequency of Antipsychotics‐Induced Tardive Dyskinesia and Other Extrapyramidal Symptoms: Analysis Based on a Spontaneous Reporting System Database in Japan
Source: Neuropsychopharmacol Rep. 2025 Sep 12;45(3):e70049. doi: 10.1002/npr2.70049 (PMC12426896; doi:10.1002/npr2.70049)
Supplement: Supplementary file 1 — Figure S1: Forest Plot of the Crude Reporting Odds Ratios for Risk Signals for Reporting Each Adverse Drug Reaction for Each Antipsychotics Category versus Non‐Antipsychotics Crude RORs with 95% CIs are plotted on a log scale. Crude RORs greater than 1 indicate higher risk signals of reporting an ADR associated with antipsychotics versus non‐antipsychotics. ADR, adverse drug reaction; CIs, confidence intervals; FGAs, first‐generation antipsychotics; RORs, reporting odds ratios; SGAs, second‐generation antipsychotics. Figure S2: Forest Plot of the Crude Reporting Odds Ratios for Risk Signals for Reporting Each Adverse Drug Reaction for Each Second‐Generation Antipsychotics Category versus First‐Generation Antipsychotics Crude RORs with 95% CIs are plotted on a log scale. Crude RORs greater than 1 indicate higher risk signals for reporting an ADR associated with SGAs versus FGAs. ADR, adverse drug reaction; CIs, confidence intervals; FGAs, first‐generation antipsychotics; RORs, reporting odds ratios; SGAs, second‐generation antipsychotics. Table S1: Classification of antipsychotics. Table S2: Two‐by‐two contingency table to calculate reporting odds ratios. Table S3: Non‐antipsychotics reported in ≥ 5 cases for tardive dyskinesia or ≥ 10 cases for extrapyramidal symptoms in alphabetical order. Table S4: Cases of adverse drug reactions of interest used to calculate reporting odds ratios using the two‐by‐two contingency table described in Table S2. [file NPR2-45-e70049-s001.docx]

Reporting Frequency of Antipsychotics-Induced Tardive Dyskinesia and other Extrapyramidal Symptoms: Analysis Based on a Spontaneous Reporting System Database in Japan

Saga et al.

# Supplementary Materials

## Table S1. Classification of Antipsychotics

| Antipsychotic | Pharmacology domain | Mode of action | Classification |
| --- | --- | --- | --- |
| Bromperidol |  | Antagonist | FGA |
| Chlorpromazine | Dopamine, serotonin | Antagonist | FGA |
| Clocapramine |  | Antagonist | FGA |
| Fluphenazine | Dopamine | Antagonist | FGA |
| Haloperidol | Dopamine | Antagonist | FGA |
| Levomepromazine |  | Antagonist | FGA |
| Mosapramine |  | Antagonist | FGA |
| Nemonapride |  | Antagonist | FGA |
| Perphenazine | Dopamine | Antagonist | FGA |
| Pimozide | Dopamine | Antagonist | FGA |
| Propericiazine |  | Antagonist | FGA |
| Sulpiride | Dopamine | Antagonist | FGA |
| Timiperone |  | Antagonist | FGA |
| Blonanserin | Dopamine, serotonin | Antagonist | SGA group 1 |
| Lurasidone | Dopamine, serotonin | Antagonist | SGA group 1 |
| Olanzapine | Dopamine, serotonin | Antagonist | SGA group 1 |
| Perospirone | Dopamine, serotonin | Antagonist | SGA group 1 |
| Zotepine | Dopamine, serotonin | Antagonist | SGA group 1 |
| Asenapine | Dopamine, serotonin, norepinephrine | Antagonist | SGA group 2 |
| Clozapine^a^ | Dopamine, serotonin, norepinephrine | Antagonist | SGA group 2 |
| Paliperidone | Dopamine, serotonin, norepinephrine | Antagonist | SGA group 2 |
| Risperidone | Dopamine, serotonin, norepinephrine | Antagonist | SGA group 2 |
| Quetiapine | Dopamine, serotonin, norepinephrine | Multimodal | SGA group 3 |
| Aripiprazole | Dopamine, serotonin | Partial agonist and antagonist | SGA group 4 |
| Brexpiprazole | Dopamine, serotonin | Partial agonist and antagonist | SGA group 4 |

^a^Clozapine cases were excluded from the analysis, because the influence on ADRs by prior use of other antipsychotics or their interactions could potentially confound the analysis.

## Table S2. Two-by-Two Contingency Table to Calculate Reporting Odds Ratios

|  | Adverse drug reactions of interest | Other adverse drug reactions (reference) | Total |
| --- | --- | --- | --- |
| Drug of interest | a | b | a + b |
| Other drugs (reference) | c | d | c + d |
| Total | a + c | b + d | a + b + c + d |

The reporting odds ratio for a drug of interest was calculated as (a/c)/(b/d) = ad/bc.^20^

## Table S3. Non-Antipsychotics Reported in ≥5 Cases for Tardive Dyskinesia or ≥10 Cases for Extrapyramidal Symptoms in Alphabetical Order

| Tardive dyskinesia (n ≥ 5) | Extrapyramidal symptoms (n ≥ 10) |
| --- | --- |
| Amoxapine | Aciclovir |
| Biperiden hydrochloride | Allopurinol |
| Brotizolam | Alprazolam |
| Chlorpromazine/promethazine combination drug | Amantadine hydrochloride |
| Clonazepam | Amlodipine besilate |
| Domperidone | Amoxapine |
| Etizolam | Bevacizumab (genetic recombination) |
| Flunitrazepam | Biperiden hydrochloride |
| Lithium carbonate | Bromazepam |
| Mirtazapine | Brotizolam |
| Nitrazepam | Calcium folinate |
| Sertraline hydrochloride | Carbamazepine |
| Tiapride hydrochloride | Ceftriaxone sodium hydrate |
| Trihexyphenidyl hydrochloride | Chlorpromazine/promethazine combination drug |
| Zopiclone | Ciclosporin |
|  | Clomipramine hydrochloride |
|  | Clonazepam |
|  | Clotiazepam |
|  | Concentrated human blood platelet |
|  | Diazepam |
|  | Domperidone |
|  | Donepezil hydrochloride |
|  | Duloxetine hydrochloride |
|  | Entacapone |
|  | Escitalopram oxalate |
|  | Ethyl loflazepate |
|  | Etizolam |
|  | Famotidine |
|  | Fentanyl citrate |
|  | Flunitrazepam |
|  | Fluorouracil |
|  | Fluphenazine maleate |
|  | Fluvoxamine maleate |
|  | Furosemide |
|  | Galantamine hydrobromide |
|  | Human papillomavirus bivalent (Types 16 and 18) vaccine, recombinant |
|  | Human papillomavirus quadrivalent (Types 6, 11, 16, 18) vaccine, recombinant |
|  | Influenza HA vaccine |
|  | Irinotecan hydrochloride hydrate |
|  | Irradiated red blood cells |
|  | Istradefylline |
|  | Itraconazole |
|  | Lacosamide |
|  | Lamotrigine |
|  | Lansoprazole |
|  | Levetiracetam |
|  | Levocetirizine hydrochloride |
|  | Levodopa |
|  | Levodopa/benserazide hydrochloride |
|  | Levodopa/carbidopa hydrate |
|  | Levodopa/carbidopa hydrate/entacapone |
|  | Levofloxacin hydrate |
|  | Lithium carbonate |
|  | Lorazepam |
|  | Loxoprofen sodium hydrate |
|  | Memantine hydrochloride |
|  | Metformin hydrochloride |
|  | Methotrexate |
|  | Metoclopramide hydrochloride |
|  | Metronidazole |
|  | Midazolam |
|  | Milnacipran hydrochloride |
|  | Mirtazapine |
|  | Mosapride citrate hydrate |
|  | Nitrazepam |
|  | Oxaliplatin |
|  | Paroxetine hydrochloride hydrate |
|  | Pembrolizumab (genetic recombination) |
|  | Perampanel hydrate |
|  | Phenytoin |
|  | Pramipexole hydrochloride hydrate |
|  | Prednisolone |
|  | Pregabalin |
|  | Prochlorperazine maleate |
|  | Ramelteon |
|  | Rasagiline mesilate |
|  | Rivastigmine |
|  | Ropinirole hydrochloride |
|  | Rotigotine |
|  | Selegiline hydrochloride |
|  | Sertraline hydrochloride |
|  | Silodosin |
|  | Sodium valproate |
|  | Suvorexant |
|  | Tacrolimus hydrate |
|  | Teriparatide acetate |
|  | Theophylline |
|  | Tiapride hydrochloride |
|  | Tramadol hydrochloride |
|  | Tramadol hydrochloride and acetaminophen |
|  | Trazodone hydrochloride |
|  | Triazolam |
|  | Trihexyphenidyl hydrochloride |
|  | Valaciclovir hydrochloride |
|  | Venlafaxine hydrochloride |
|  | Vidarabine |
|  | Voriconazole |
|  | Zolpidem tartrate |
|  | Zonisamide |
|  | Zopiclone |

## Table S4. Cases of Adverse Drug Reactions of Interest Used to Calculate Reporting Odds Ratios Using the Two-by-Two Contingency Table Described in Table S2

| ADR of interest  Antipsychotics category | a | a' | b | b' | c | c' | d | d’ |
| --- | --- | --- | --- | --- | --- | --- | --- | --- |
| Tardive dyskinesia (n) |  |  |  |  |  |  |  |  |
| FGAs | 154 | – | 4,124 | – | 259 | – | 1,067,421 | – |
| SGAs total | 323 | 323 | 13,968 | 13,968 | 259 | 154 | 1,067,421 | 4,124 |
| SGA group 1 | 118 | 118 | 3,248 | 3,248 | 259 | 154 | 1,067,421 | 4,124 |
| SGA group 2 | 90 | 90 | 5,007 | 5,007 | 259 | 154 | 1,067,421 | 4,124 |
| SGA group 3 | 51 | 51 | 2,380 | 2,380 | 259 | 154 | 1,067,421 | 4,124 |
| SGA group 4 | 64 | 64 | 3,333 | 3,333 | 259 | 154 | 1,067,421 | 4,124 |
| Akathisia (n) |  |  |  |  |  |  |  |  |
| FGAs | 47 | – | 4,124 | – | 207 | – | 1,067,421 | – |
| SGAs total | 125 | 125 | 13,968 | 13,968 | 207 | 47 | 1,067,421 | 4,124 |
| SGA group 1 | 37 | 37 | 3,248 | 3,248 | 207 | 47 | 1,067,421 | 4,124 |
| SGA group 2 | 51 | 51 | 5,007 | 5,007 | 207 | 47 | 1,067,421 | 4,124 |
| SGA group 3 | 12 | 12 | 2,380 | 2,380 | 207 | 47 | 1,067,421 | 4,124 |
| SGA group 4 | 25 | 25 | 3,333 | 3,333 | 207 | 47 | 1,067,421 | 4,124 |
| Dyskinesia (n) |  |  |  |  |  |  |  |  |
| FGAs | 85 | – | 4,124 | – | 885 | – | 1,067,421 | – |
| SGAs total | 158 | 158 | 13,968 | 13,968 | 885 | 85 | 1,067,421 | 4,124 |
| SGA group 1 | 52 | 52 | 3,248 | 3,248 | 885 | 85 | 1,067,421 | 4,124 |
| SGA group 2 | 44 | 44 | 5,007 | 5,007 | 885 | 85 | 1,067,421 | 4,124 |
| SGA group 3 | 28 | 28 | 2,380 | 2,380 | 885 | 85 | 1,067,421 | 4,124 |
| SGA group 4 | 34 | 34 | 3,333 | 3,333 | 885 | 85 | 1,067,421 | 4,124 |
| Dystonia (n) |  |  |  |  |  |  |  |  |
| FGAs | 125 | – | 4,124 | – | 368 | – | 1,067,421 | – |
| SGAs total | 359 | 359 | 13,968 | 13,968 | 368 | 125 | 1,067,421 | 4,124 |
| SGA group 1 | 108 | 108 | 3,248 | 3,248 | 368 | 125 | 1,067,421 | 4,124 |
| SGA group 2 | 107 | 107 | 5,007 | 5,007 | 368 | 125 | 1,067,421 | 4,124 |
| SGA group 3 | 52 | 52 | 2,380 | 2,380 | 368 | 125 | 1,067,421 | 4,124 |
| SGA group 4 | 92 | 92 | 3,333 | 3,333 | 368 | 125 | 1,067,421 | 4,124 |
| Parkinsonism (n) |  |  |  |  |  |  |  |  |
| FGAs | 169 | – | 4,124 | – | 436 | – | 1,067,421 | – |
| SGAs total | 244 | 244 | 13,968 | 13,968 | 436 | 169 | 1,067,421 | 4,124 |
| SGA group 1 | 66 | 66 | 3,248 | 3,248 | 436 | 169 | 1,067,421 | 4,124 |
| SGA group 2 | 92 | 92 | 5,007 | 5,007 | 436 | 169 | 1,067,421 | 4,124 |
| SGA group 3 | 32 | 32 | 2,380 | 2,380 | 436 | 169 | 1,067,421 | 4,124 |
| SGA group 4 | 54 | 54 | 3,333 | 3,333 | 436 | 169 | 1,067,421 | 4,124 |
| Parkinsonian gait (n) |  |  |  |  |  |  |  |  |
| FGAs | 1 | – | 4,124 | – | 18 | – | 1,067,421 | – |
| SGAs total | 5 | 5 | 13,968 | 13,968 | 18 | 1 | 1,067,421 | 4,124 |
| SGA group 1 | 1 | 1 | 3,248 | 3,248 | 18 | 1 | 1,067,421 | 4,124 |
| SGA group 2 | 0 | 0 | 5,007 | 5,007 | 18 | 1 | 1,067,421 | 4,124 |
| SGA group 3 | 0 | 0 | 2,380 | 2,380 | 18 | 1 | 1,067,421 | 4,124 |
| SGA group 4 | 4 | 4 | 3,333 | 3,333 | 18 | 1 | 1,067,421 | 4,124 |
| Bradykinesia (n) |  |  |  |  |  |  |  |  |
| FGAs | 2 | – | 4,124 | – | 28 | – | 1,067,421 | – |
| SGAs total | 17 | 17 | 13,968 | 13,968 | 28 | 2 | 1,067,421 | 4,124 |
| SGA group 1 | 5 | 5 | 3,248 | 3,248 | 28 | 2 | 1,067,421 | 4,124 |
| SGA group 2 | 6 | 6 | 5,007 | 5,007 | 28 | 2 | 1,067,421 | 4,124 |
| SGA group 3 | 1 | 1 | 2,380 | 2,380 | 28 | 2 | 1,067,421 | 4,124 |
| SGA group 4 | 5 | 5 | 3,333 | 3,333 | 28 | 2 | 1,067,421 | 4,124 |
| Dysarthria (n) |  |  |  |  |  |  |  |  |
| FGAs | 4 | – | 4,124 | – | 200 | – | 1,067,421 | – |
| SGAs total | 12 | 12 | 13,968 | 13,968 | 200 | 4 | 1,067,421 | 4,124 |
| SGA group 1 | 4 | 4 | 3,248 | 3,248 | 200 | 4 | 1,067,421 | 4,124 |
| SGA group 2 | 3 | 3 | 5,007 | 5,007 | 200 | 4 | 1,067,421 | 4,124 |
| SGA group 3 | 2 | 2 | 2,380 | 2,380 | 200 | 4 | 1,067,421 | 4,124 |
| SGA group 4 | 3 | 3 | 3,333 | 3,333 | 200 | 4 | 1,067,421 | 4,124 |
| Anarthria (n) |  |  |  |  |  |  |  |  |
| FGAs | 10 | – | 4,124 | – | 580 | – | 1,067,421 | – |
| SGAs total | 26 | 26 | 13,968 | 13,968 | 580 | 10 | 1,067,421 | 4,124 |
| SGA group 1 | 3 | 3 | 3,248 | 3,248 | 580 | 10 | 1,067,421 | 4,124 |
| SGA group 2 | 14 | 14 | 5,007 | 5,007 | 580 | 10 | 1,067,421 | 4,124 |
| SGA group 3 | 5 | 5 | 2,380 | 2,380 | 580 | 10 | 1,067,421 | 4,124 |
| SGA group 4 | 4 | 4 | 3,333 | 3,333 | 580 | 10 | 1,067,421 | 4,124 |
| Cogwheel rigidity (n) |  |  |  |  |  |  | |  |
| FGAs | 0 | – | 4,124 | – | 2 | – | 1,067,421 | – |
| SGAs total | 0 | 0 | 13,968 | 13,968 | 2 | 0 | 1,067,421 | 4,124 |
| SGA group 1 | 0 | 0 | 3,248 | 3,248 | 2 | 0 | 1,067,421 | 4,124 |
| SGA group 2 | 0 | 0 | 5,007 | 5,007 | 2 | 0 | 1,067,421 | 4,124 |
| SGA group 3 | 0 | 0 | 2,380 | 2,380 | 2 | 0 | 1,067,421 | 4,124 |
| SGA group 4 | 0 | 0 | 3,333 | 3,333 | 2 | 0 | 1,067,421 | 4,124 |
| Tremor (n) |  |  |  |  |  |  |  |  |
| FGAs | 24 | – | 4,124 | – | 1074 | – | 1,067,421 | – |
| SGAs total | 65 | 65 | 13,968 | 13,968 | 1074 | 24 | 1,067,421 | 4,124 |
| SGA group 1 | 14 | 14 | 3,248 | 3,248 | 1074 | 24 | 1,067,421 | 4,124 |
| SGA group 2 | 23 | 23 | 5,007 | 5,007 | 1074 | 24 | 1,067,421 | 4,124 |
| SGA group 3 | 12 | 12 | 2,380 | 2,380 | 1074 | 24 | 1,067,421 | 4,124 |
| SGA group 4 | 16 | 16 | 3,333 | 3,333 | 1074 | 24 | 1,067,421 | 4,124 |
| Akinesia (n) |  |  |  |  |  |  |  |  |
| FGAs | 7 | – | 4,124 | – | 84 | – | 1,067,421 | – |
| SGAs total | 24 | 24 | 13,968 | 13,968 | 84 | 7 | 1,067,421 | 4,124 |
| SGA group 1 | 10 | 10 | 3,248 | 3,248 | 84 | 7 | 1,067,421 | 4,124 |
| SGA group 2 | 10 | 10 | 5,007 | 5,007 | 84 | 7 | 1,067,421 | 4,124 |
| SGA group 3 | 1 | 1 | 2,380 | 2,380 | 84 | 7 | 1,067,421 | 4,124 |
| SGA group 4 | 3 | 3 | 3,333 | 3,333 | 84 | 7 | 1,067,421 | 4,124 |
| Hypersalivation (n) |  |  |  |  |  |  |  |  |
| FGAs | 8 | – | 4,124 | – | 90 | – | 1,067,421 | – |
| SGAs total | 42 | 42 | 13,968 | 13,968 | 90 | 8 | 1,067,421 | 4,124 |
| SGA group 1 | 12 | 12 | 3,248 | 3,248 | 90 | 8 | 1,067,421 | 4,124 |
| SGA group 2 | 18 | 18 | 5,007 | 5,007 | 90 | 8 | 1,067,421 | 4,124 |
| SGA group 3 | 4 | 4 | 2,380 | 2,380 | 90 | 8 | 1,067,421 | 4,124 |
| SGA group 4 | 8 | 8 | 3,333 | 3,333 | 90 | 8 | 1,067,421 | 4,124 |

The numbers of cases with a–d were used for the analysis of antipsychotics vs. non-antipsychotics, and the numbers with a’–d’ were used for the analysis of SGAs (total or each group) vs. FGAs.

ADR: adverse drug reaction; FGAs: first-generation antipsychotics; SGAs: second-generation antipsychotics.

## Figure S1. Forest Plot of Crude Reporting Odds Ratios for Risk Signals for Reporting Each Adverse Drug Reaction for Each Antipsychotics Category vs. Non-Antipsychotics

Crude RORs with 95% CIs are plotted on a log scale. Crude RORs greater than 1 indicate higher risk signals of reporting an ADR associated with antipsychotics vs. non-antipsychotics.

ADR: adverse drug reactions; CIs: confidence intervals; FGAs: first-generation antipsychotics; RORs: reporting odds ratios; SGAs: second-generation antipsychotics.





## Figure S2. Forest Plot of Crude Reporting Odds Ratios for Risk Signals for Reporting Each Adverse Drug Reaction for Each Second-Generation Antipsychotics Category vs. First-Generation Antipsychotics

Crude RORs with 95% CIs are plotted on a log scale. Crude RORs greater than 1 indicate higher risk signals for reporting an ADR associated with SGAs vs. FGAs.

ADR: adverse drug reactions; CIs: confidence intervals; FGAs: first-generation antipsychotics; RORs: reporting odds ratios; SGAs: second-generation antipsychotics.
